# Analysis of apo and citraconate-bound hACOD1 (hIRG1) by X-ray crystallography and NMR spectroscopy: Structural insights for developing chemotherapeutic agents

**DOI:** 10.1101/2025.06.13.659517

**Published:** 2025-06-13

**Authors:** Brent Runge, Ian J. Fucci, Diana C. F. Monteiro

**Affiliations:** 1 Center for Structural Biology, Center for Cancer Research, National Cancer Research, National Institutes of Health, Frederick, MD 21702 USA

## Abstract

In this work, we present the high-resolution structure of human aconitate decarboxylase 1 (hACOD1) in its true apo form (active site empty) as well in complex with the inhibitor citraconate. These two new structures show the architecture of the active site and the structure-activity relationships of citraconate inhibition. Careful analysis of the structures indicates probable dynamics required for substrate/inhibitor binding and catalysis. These observations were further explored using molecular dynamic simulations, which show a clear open-close mechanism of hACOD1 between the A1 and A2 loops, the lid- and helical-domain respectively. As part of the biochemical characterization of the protein, we also developed an alternative kinetic assay which measures the rate of catalysis of hACOD1 by direct observation of the conversion of *cis*-aconitate to itaconate by NMR spectroscopy. The work herein offers a foundation for structure- and dynamic-driven design of novel hACOD1 inhibitors as novel chemotherapeutics.

## 1. Introduction

**Fig. 1:**
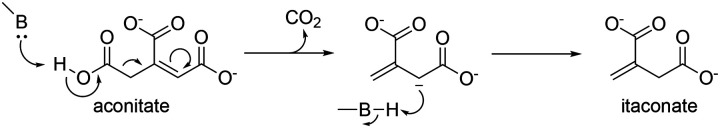
Reaction mechanism of cis-aconitate decarboxylation by ACOD1.

Human aconitate decarboxylase 1 (hACOD1) plays a pivotal immunoregulatory role in host-pathogen interactions^1–4^, both pro- and anti-inflammatory responses^5–7^, and modulates downstream mechanisms regulating oxidative stress^8,9^. Moreover, hACOD1 has been shown as a significant contributor to the maintenance of the tumor microenvironments^10,11^. hACOD1 is encoded by the human immune regulatory gene 1 (hIRG1) and is responsible for catalyzing the production of itaconate from *cis*-aconitate (Fig.1). Itaconate was initially identified as a key metabolite upregulated in macrophages as a response to bacterial infections triggered by recognition of pathogen-associated molecular patterns (PAMPS), including lipopolysaccharides (LPS)^12^. It was later linked to the now known aconitate decarboxylase activity within the tricarboxylic acid (TCA) cycle Michelucci *et. al*^13^.

Itaconate has a direct regulatory effect on the TCA cycle, by inhibiting succinate dehydrogenase (SDH), upregulating succinate concentration in cells, which impacts mitochondrial respiration and cytokine production in macrophages^6^. These regulatory mechanisms lead to anti-inflammatory responses including halting of mitochondrial reactive oxygen species production and downregulation of HIF-1a activity and IL-1b expression. Itaconate also further regulates antioxidant responses by modulation of KEAP1, stopping proteasomal degradation of the transcription factor NRF2^14^. The increase in NRF2 leads to ROS detoxification and protects cells against cytotoxic oxidative stress^15^. All these anti-inflammatory cascades, resulting from increased itaconate production, effectively lead to a cellular environment more resilient to cytotoxic conditions and thus resistant to apoptotic mechanisms.

hACOD1 has been shown to have elevated expression levels in cancer cells and is a reported tumor growth factor^10,11,16,17^. This observation, together with the current understanding of the role of itaconate in cancer cell metabolism, makes hACOD1 a promising chemotherapeutic target for sensitizing cancer cells and elicit ferroptosis^18^. Thus, combination therapies involving the inhibition of hACOD1 may circumvent chemotherapeutic resistant tumor phenotypes. Currently, citraconate - a structural mimetic of the substrate *cis*-aconitate - has been shown to inhibit hACOD1 activity by competitively binding with aconitate to the active site^19^. But the structure of the hACOD1-citraconate complex has so far evaded elucidation. The biochemical and biophysical characterization of hACOD1 has been scarce compared to the extensive cellular and metabolic profiling of this enzyme. Obtaining the hACOD1-citraconate complex structure allows for the elucidation of the structure-activity relationships associate with inhibition and open the doors to novel inhibitor design. By designing novel hACOD1 inhibitors, these can be used as therapeutic agents balancing the tumor microenvironment, and reducing cellular proliferation and resilience, thus increasing the therapeutic window and efficacy of chemotherapeutic agents when used as dual therapies.

Here we present a comprehensive structural and catalytic investigation of hACOD1 and its interactions with citraconate, along with a description of specific protein crystallization decisions that allowed for the solution of a true apo structure of the enzyme and subsequent inhibitory complex structure. As part of this work, we also explored the dynamics intrinsic to substrate binding and release, vital for the design of novel potent inhibitors beyond citraconate.

## 2. Results and discussion

### 2.1. An artifact-free structure of apo ACOD1

Protein crystallization typically requires the use of chemical cocktails that drive the formation of specific protein-protein contacts while pushing the protein beyond its solubility limit. Successful crystallization of proteins requires the empirical identification of buffer components and specific precipitants that drive successful crystal growth over amorphous precipitation. Crystallization cocktails typically screen for optimal buffers, including pH ranges, protein precipitation agents such as polyethylene glycols (PEGs) or ammonium sulfate, inorganic salts and additives. Polydentate carboxylates (i.e. citrate or tacsimate) are common crystallization components found in sparse matrix screening kits. Since the substrate of hACOD1 is a polydentate carboxylate, it is unsurprising that the previously published structures of hACOD1 and murine ACOD1 (mACOD1) were obtained from crystals grown in sodium citrate and tacsimate respectively^20^. But these polydentate carboxylates can mimic the enzyme’s natural substrate and compete with protein binders for the active site, making it difficult to obtain a proteinligand structure from crystals grown in conditions where citrate is several orders of magnitude more concentrated than substrate. Accordingly, the previously reported hACOD1 structure shows evidence of unstructured crystallization additives in the active site.

**Fig. 2:**
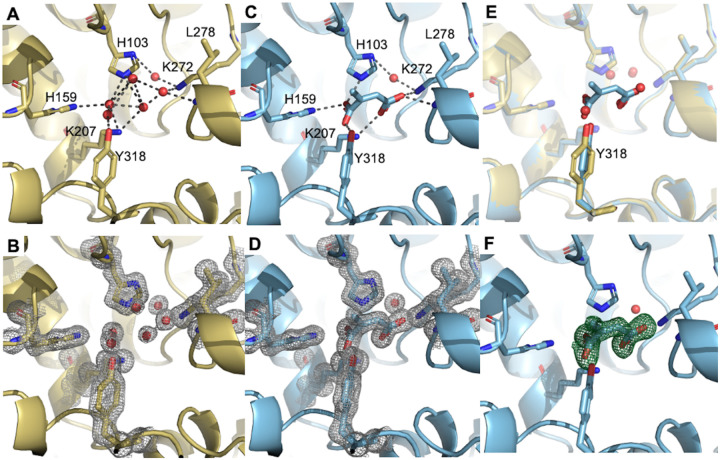
Crystal structures of apo hIRG (yellow, A&B) and citraconate-bound hACOD1 (blue, C&D), showing relevant residues in the active site. A) Apo hACOD1 showing the hydrogen-bonding network between residues lining the active site and ordered waters (black dashes) and B) the 2Fo-Fc electron density map contoured at 1σ (gray mesh). C) Citraconate-bound hACOD1 showing the hydrogen-bonding network between the inhibitor and active site residues and D) the 2Fo-Fc electron density map contoured at 1σ (gray mesh). E) Overlay of apo and citraconate-bound hACOD1, showing minimal movement of residues in the active site, except for Tyr318 which is partly rotated. The waters in the apo form sit in positions occupied by oxygens of the citraconate carboxylates. F) Citraconate-removed omit map, showing clear density for the ligand.

After optimizing the expression and purification of hACOD1 to yield stable, active and pure protein suitable for structural and enzymatic studies (see methods and table S1 for details), we set out to obtain reliable crystallization conditions for hACOD1 that yield well-diffracting crystals that could be used when driving a structure-based medicinal chemistry campaign to develop novel hACDO1 inhibitors. We obtained several crystallization hits from sparse matrix screening (576 conditions), most of which contained unwanted polydentate carboxylates. From these, 11 conditions (Sup. Table 3) were chosen for further optimization and crystals diffracting routinely to 1.3 Å or better were obtained from conditions containing calcium acetate. Upon structure solution, no evidence of bound acetate was found in the active site, indicating that our crystallization pipeline had been successful in producing a true apo structure (Fig. 2, A and B).

### 2.2. Structural comparison of apo-from and citraconate-bound ACOD1

Both apo hACOD1 and citraconate-bound hACOD1 crystallized in similar conditions and diffracted to high resolution (see Sup. Table 1 for crystallization details, Sup. Table 4 for data reduction statistics and Table 1 for data refinement statistics). Data were collected to very high resolution, at 1.32 Å and 1.22 Å for the citraconate-bound and apo forms respectively. Fig. 2, A and C show the active site architecture of hACOD1 with and without citraconate, including electron density maps for the relevant residues and water molecules (B and D). The pose of the citraconate molecule was further corroborated by generating an omit map (a map calculated after removal of the ligand to show unbiased presence of electron density for it, Fig. 2, F), which returns perfectly matching density for citraconate.

The RMSD between the two structures is very low, at 0.097 Å (0.108 Å between same chains), indicating a consistent fold and conformation that does not differ between the apo and ligated forms. The apo protein shows a robust water network in the active site, with hydrogen bonds to residues His103, His159, Lys207, Lys272, Tyr318 and the backbone of Leu278 (Fig. 2, A). Binding of citraconate displaces four ordered waters visible in the empty active site (Fig. 2, C), all of which occupy positions exactly matching those of the carboxylate oxygens in the citraconate ligand (Fig. 2, E). From all the residues comprising the active site, only Tyr318 shows a positional change between the two protein forms (Fig. 2, E). The sidechain of Tyr318 moves by 1.7 Å to switch between hydrogen bonding to water or citraconate. Otherwise, the active site is virtually identical, with the substrate binding pocket shielded from the bulk solvent by Tyr318. This indicates that the protein must undergo specific dynamic motions to allow for binding and release of substrates and products and that these dynamics could be used to generate new inhibitor scaffold beyond substrate mimetics. Fig. 5, B shows the active site cavity and its location relative to the protein surface. The two solvent exposed areas are shielded by a small number of residues, with Tyr318 making part of this interface. Movement of this residue, and associated loop, should allow for access to the active site, as proposed by molecular dynamics (see section 2.4).

hACOD1 activity follows a typical acid-base catalytic mechanism^21^, but the identity of the base, or bases, involved in the proton transfer mechanism is still unknown. The active site is well-defined and previous work based on crystal structures of murine and human ACOD1, and prior works indicates that His103 should be involved due to its proximity^20^. Our structure shows that indeed His103 is well position abstract a proton at the C2 position of citraconate, corroborating the base-catalyzed decarboxylation.

### 2.3. Analysis of ACOD1 enzymatic activity by NMR-based assay

**Fig. 3:**
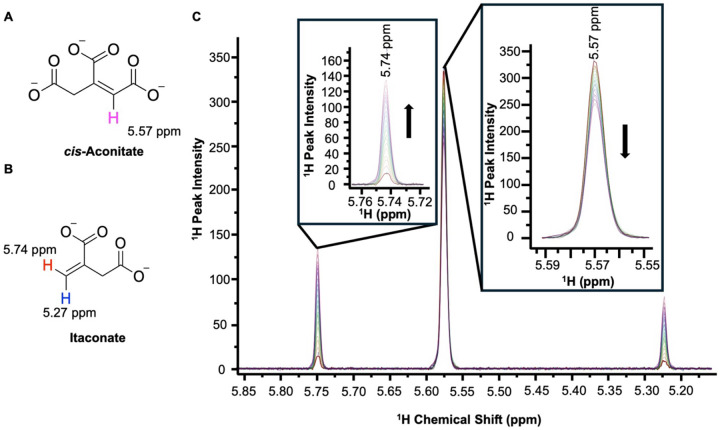
NMR-based assay of hACOD1 activity, showing typical progression ^1^H NMR traces to follow the conversion of *cis*-aconitate to itaconate over a 2-hour time course. (A) Structure of cis-aconitate with alkene proton highlighted in pink (~5.57 ppm). (B) Structure of Itaconate with alkene protons highlighted in red (~5.74 ppm) and blue (~5.27 ppm) to distinguish most upfield and downfield ^1^H peaks. (C) ^1^H NMR spectra of hACOD1 kinetic assay over a two-hour time course.

To observe the inhibitory effects of citraconate and future hACOD1 inhibitors, an NMR-based catalytic assay was designed which observes both the production itaconate and concurrent depletion of *cis*-aconitate. Based on current models of decarboxylation of *cis*-aconitate by hACOD1^21^, deprotonation of the carboxyl group (C5) is facilitated by a basic moiety in the active site with the release of CO_2_ resulting in the subsequent protonation of the carbanion intermediate (Fig. 1). The conversion of the tri-substituted alkene of *cis*-aconitate (Fig. 3, A) to a 1,1- or geminal position of the two carboxylate moieties (Fig. 3, B) produces distinct alkene signals quantifiable by ^1^H NMR (Fig. 3, C). The assay was conducted by measuring the relative intensity of the loss or evolution of alkene ^1^H peaks corresponding cis-aconitate and itaconate respectively. Time-course experiments were recorded with spectra taken every six minutes for two hours to determine the reaction progress. The initial rate for each kinetic assay was derived by linear regression of the peak intensities with respect to time for the initial 10% turnover of *cis*-aconitate. Given the temperature-dependence of ^1^H chemical shifts, we found that temperature equilibration of samples prior to adding protein to solution was necessary to extract accurate measurements.

**Fig. 4:**
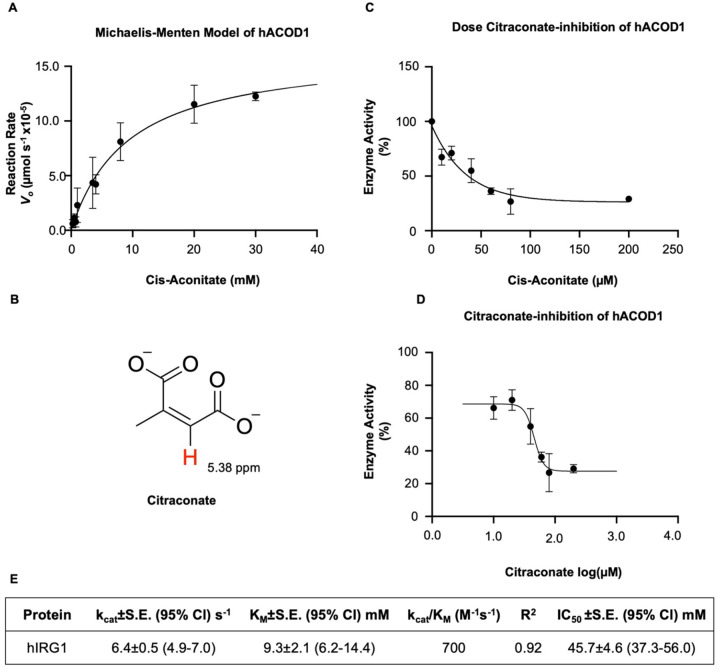
Analysis of hACOD1 enzymatic activity. (A) Michaelis-Menten plot of cis-aconitate decarboxylation by hACOD1. (B) Structure of citraconate with alkene proton highlighted (C) Dose-dependent inhibition plot of hACOD1 (D) Dose-dependent response curve. (E) Kinetic and inhibition constants for hACOD1.

Running these experiments continuously as opposed to as an endpoint assay allowed us to obtain the initial reaction rates by varying *cis*-aconitate concentration from 0.25 to 30 mM (Fig. 4, A). By using Michaelis-Menten kinetics, we determined the catalytic turnover of hACOD1 to be (k_cat_= 6.4±0.5 s^−1^). This value is higher than that previously reported (k_cat_= 0.94±0.02 s^−1^) and closer to that previously reported for mACOD1 (k_cat_= 4.94±0.13 s^−1^)^20^. Moreover, the derived K_M_ of hACOD1 (9.3±2.1 mM) closer to that of the *Aspergillus* protein (K_M_=9.0±1.7 mM) and deviated from previously reported kinetics of murine (K_M_=0.65±0.08) and human ACOD1 (K_M_=0.61±0.06 mM)^20^. We speculate that this observed increase in protein activity may be due to the use of a continuous assay as opposed to an endpoint assay (i.e. HPLC) and/or differences in expression, purification, storage protocols.

To determine the inhibition kinetics of hACOD1 by citraconate we performed a series of NMR assays in the presence of increasing inhibitor concentration. While similar in structure, the tri-substituted scaffolding of citraconate produces a unique alkene ^1^H chemical shift (^1^Hδ~5.38 ppm) distinct from evolving itaconate peaks (^1^Hδ ~5.54 and 5.27 ppm, Sup. Fig. 2). Like the assays preformed on apo-protein, two-hour time-course experiments were used to measure reduction in hACOD1 enzymatic activity (Fig. 4, C and D). The *in vitro* inhibitory effect measured in this study (IC_50_=45.7 μM) is comparable to that previously determined by cellular studies^19^.

### 2.4. Protein dynamics and catalysis

**Fig. 5:**
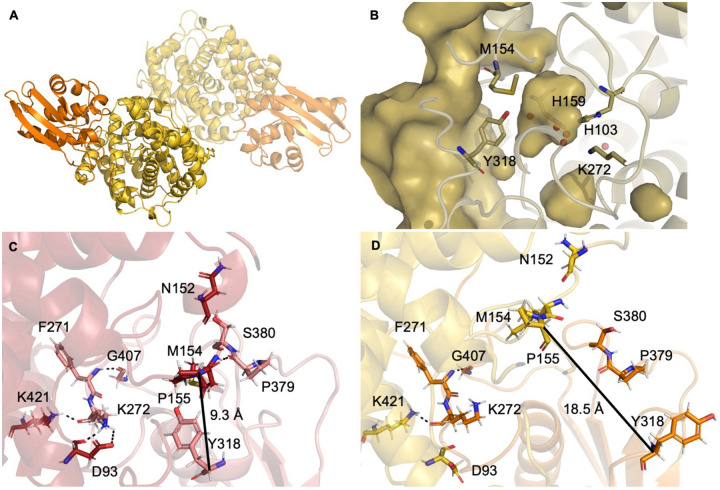
Architecture of hACOD1. A) quaternary structure of hACOD1, showing the biologically relevant dimer, with the two monomers represented at different transparencies. The two domains, lid and alpha-helical are represented as orange and yellow respectively. B) surface representation of the closed form of hACOD1, showing the small, closed active site cavity and the proposed gating residues, Tyr318 and Met154, separating the active site from the external surface. C) and D) the closed (red/pink) and open (yellow/orange) conformations of hACOD1 obtained from MD simulations. Residues making inter-domain hydrogen bonds in the closed conformation (C) are shown in sticks with dashed black lines for the bonds. The same residues and remaining hydrogen bonds are shown for the open conformation (D). The distance between the Cα of Tyr318 and Met154 is calculated for both conformations and displayed as a black, solid line.

hACOD1 is a dimer, with each protomer composed of two well-folded domains, a liddomain composed of residues 273–410 and an alpha-helical domain, residues 1–267 and 413–461 (Fig. 5, A), matching the typical topology of the MmgE/PrpD superfamily (EMBL-EBI InterPro). The catalytic sites are located between the two domains, with catalytically relevant residues His103, His159 and Lys 272 in the alpha-helical domain and Tyr318 in the lid domain. With the active site presenting as closed in both our apo- and citraconate-bound structures, a question remained regarding the mechanism of substrate (and inhibitor) binding, which must require the opening and closing of a ligand channel^22^.

Contrary to the large interface between the two monomers, there are only a small number of inter-domain hydrogen bonds, specifically between Met154-Pro379, Asn152-Ser380, Lys272-Asp93/Lys421 and Gly407-Phe271 (Fig. 5, C). The loop containing Tyr318 is poised in a perfect position to afford the opening of a channel to the active site (Fig. 5, B). Molecular dynamic simulations of the dimeric hACOD1 show a clear open-closed equilibrium involving the lid and alpha-helical domains. This motion results in a large conformational change of the active site with the linchpin consisting of residues Tyr318 (lid domain), Met154, Pro155 and Met199 (α-helical domain) releasing, allowing the lid to open and granting access for substrate to bind (Fig. 5, C and D). The linchpin is formed by hydrophobic interactions between the aromatic ring of Tyr318 and the methionine residues, leaving the tyrosine hydroxyl free to engage with substrate, as illustrated by the citraconate-bound structure (Fig. 2). When aggregated over all 2 μs of simulation, the distribution of interatomic distances between Cα of Tyr318 and of Pro155 is bimodal, indicating distinct open (>13.6 Å) and closed (~10 Å) conformations. In our simulations, the system only opens seldomly, with the population representing the open conformation corresponding to ~5% of the total states (Sup. Fig. 1, A). To further elucidate the local dynamics involved in the opening mechanism, the mobility of the residues was investigated by calculating root-mean square fluctuations (RMSF). Throughout all the simulations, only one of the two protein chains (chain B) underwent opening, with chain A remaining closed. Though not a mechanistically relevant observation, this disparity between chains allowed us to compare the RMSF amplitudes between open and closed conformations. RMSF amplitude differences between chains A and B (Sup. Fig. 1, B) show that two loops spanning residues involved in the domain interface (A1 and A2, residues 147–155 and 373–387 respectively) are more mobile in the opening protomer (chain B). These observations match the structural analysis described above. Moreover, comparison of the different protein conformations reveals a ~2280 Å^2^ difference in the solvent-accessible area observed between the open- and closed-state of the ACOD1.

## 3. Conclusion

To gain structural insights to further development of citraconate-derivative inhibitors we used robust protein biochemical and crystallization procedures to obtain the first true apo- and citraconate-bound structures of hACOD1. The structures were solved to very high resolution and provide insight into how to successfully obtain ligated hACOD1 structures, a vital step in structure-activity drug design. Our citraconate-bound structure reveals that His103 is proximal to the C2 position of the inhibitor, suggesting base-catalyzed decarboxylation at this position. We observed a very structurally stable active site, with the single Tyr318 residue shifting by 1.7 Å between the apo- and ligated forms of the protein. We proposed that residue Tyr318 could be involved in the dynamics necessary to bind and release substrates and products during hACOD1 catalysis and investigated these using MD simulations.

We also designed a new enzymatic assay that directly follows the decarboxylation of *cis*-aconitate in a continuous manner. With it, we were able to reliably determine the dose-dependent inhibition of hACOD1 by citraconate. The present investigation has yielded optimal co-crystallization conditions of hACOD1 and a new unambiguous biological assay offering a complementary method for guiding the design of novel chemotherapeutics.

The dynamics characterized by MD provide evidence to support the development of inhibitors to target the protein beyond the small active site. The open-close mechanism between the lid and helical-domain depicted by our simulations demonstrates a more plastic binding cavity which can be targeted by larger molecules as well as the possibility of developing bifunctional drugs, such as PROTACS.

## 4. MATERIALS AND METHODS

### 4.1. Protein expression

The expression plasmid pCAD29_hIRG1_4–461_pvp008 was a gift from Konrad Buessow (Addgene plasmid # 124843; http://n2t.net/addgene:124843; RRID:Addgene_124843)^20^. The plasmid codes for a truncated version of ACOD1, lacking short unstructured N- and C-terminal motifs. *Escherichia coli* BL21-CodonPlus (RIPL) cells (Novagen) were used for protein expression. hACOD1 (4–461) was expressed and purified as reported previously with modifications. In brief, transformed BL21-CodonPlus (RIPL) cells (Novagen) cells were cultured in 5–10 mL of Luria-Bertani (LB) medium supplemented with 50 μg/mL kanamycin and 30 μg/mL chloramphenicol. The LB pre-culture was incubated at 37 °C with shaking at 200 rpm until the OD_600_ reached 1.2–1.5 and 1 mL of this pre-culture was used to inoculate 50 mL of LB medium. After overnight growth at 37 °C, the small LB culture was transferred to 1 L of LB medium, incubated at 37 °C with shaking at 200 rpm up to an OD_600_ of 0.75–0.80. At that point, the temperature and agitation were lowered to 22 °C and 130 rpm, respectively. After ~1 hour, protein expression was induced with isopropylthio-β-galactoside (IPTG), at a final concentration of 500 μM. Cells were grown at 22 °C for 18 hours and harvested at 6000 × *g* for 10 min at 4 °C. Cell pellets were harvested and stored at −80 °C until further use.

### 4.2. Protein purification

Frozen cell pellets were resuspended in 50 mL of wash buffer (20 mM Tris-HCl pH 8.0, 500 mM NaCl, 10% glycerol, 1 mM DTT) per 20 mg of cells. The resuspension was treated with a final concentration of 1mg/mL lysozyme, 0.01 mg/mL RNAse, and 0.005 mg/mL DNAse and the cells disrupted by sonication (*Fisherbrand Qsonica 505*) at 60% power for 5 minutes (10 s pulse on and 20 s pulse off) on ice. The cellular lysate was clarified with 6–8 mL of 10% PEI followed by centrifugation at 16,500 × g for 1 hour at 4 °C. After clarification of cellular lysate, the supernatant was loaded onto a StrepTactin column (HiTrap Q FF 5 mL, GE Healthcare). Prior to elution step, columns were washed with 5 cv of buffer A or until UV absorbance returned to baseline. Proteins were eluted using wash buffer supplemented with 500 mM Biotin. The purified protein was pooled and dialyzed overnight in wash buffer overnight at 4 °C with 0.5 mg of TEV protease to cleave His6-tag. The protein was concentrated (3–4 mL) and purified to homogeneity by size-exclusion chromatography (HiLoad 16/600 Superdex 200 prep grade, GE Healthcare) by elution with SEC buffer (10 mM HEPES, 150 mM NaCl, 10% (v/v) glycerol, 0.1 mM TCEP, pH 7.5). For X-ray diffraction and solution NMR, purified protein was concentrated up to 120 μM (6 mg/mL) and flash-frozen in small aliquots in liquid nitrogen (−180°C) for storage at −80 °C.

### 4.3. Protein crystallization screening and optimization

Initial crystallization conditions for hACOD1 were obtained from 6 sparse matrix crystallization screens (JSCG+ and Structure I+II HTS - Molecular Dimensions - and Wizard HTS, PACT HT, PEG/ION, and INDEX HT- Hampton Research). Screening was performed at room temperature (~20 °C) using sitting drop vapor diffusion in MRC3 crystallization plates (SWISSCI) using 200 nL total volumes and varying the protein to ML concentration in 1:1, 2:1 and 1:2 v/v ratios for each condition and constant 40 μL reservoir volume. The drops were set using the Formulatrix NT8 drop setter, at a constant humidity of 80%. The plates were stored and imaged at 22 °C using a Fomulatrix R1000 imager, equipped with bright field and UV fluorescence capabilities. From the observed protein crystals, 11 hits were identified as devoid of polydentate carboxylate components.

Two conditions were chosen for optimization: condition 1) 100 mM Tris (pH 8.8), 0.2 mM CaAc, 35% PEG4000; and condition B) 200 mM NaF, 35% PEG4000. Optimization was performed by varying the crystallization components concentrations and pH using an automated formulating system (FORMULATOR, Formulatrix) in the same tray layout described above. Co-crystallization screening of citraconate with ACOD1 was performed by varying the citraconate concentration between 3 and 25 mM to reach 90.0–99.0% saturation of drug based on published K_M_. Fully grown crystals of both apo ACOD1 and citraconate-bound ACOD1 were obtained in 4–6 days.

Drops containing crystals were opened under a stream of humidified air (Watershed, Mitegen) and layered with LV cryoOil (Mitegen) as a cryoprotectant, looped and vitrified in liquid nitrogen.

### 4.4. Data collection and reduction and structure solution

Data for the apo structure and citraconate-bound structure were collected at beamlines 17-ID-2 (FMX)^23^ and 17-ID-1 (AMX)^24^ respectively in fully automated mode. Parameters for data collection can be found in Sup. Table 4. The X-ray images were indexed, integrated, scaled and merged using the automated autoproc pipeline (Global Phasing)^25^ available at NSLSII. Datasets to process further were determined from the statistics obtained from the automated data reduction pipeline.

The chosen datasets were processed as follows: the output of the XDS^26^ INTEGRATE step from autoproc was parsed through pointless (within Aimless^27^, CCP4i2^28^) for space group determination and scaling. The unmerged pointless output mtz file was further reduced in StarANISO^29^ (Global Phasing) to yield an anisotropic resolution cut off. The merged file was imported back into CCP4i2, solved by molecular replacement using MOLREP^30^ with initial model PDB 6R6U^20^. Rounds of manual model building using Coot^31^ and refinement using Refmac5^32^ were performed in CCP4i2. Refinement was performed using automated TLS parameters and with an optimized, fixed X-ray weight term. TLS groups were used for refinement instead of anisotropic b-factors as these caused clear overfitting, with a widening gap between Rfree and Rcryst and no significant decrease in Rfree. For the citraconate-bound structure, a similar protocol was used, with a citraconate coordinate file and dictionary obtained from the PDB, as part of the coot library, with monomer code CIZ.

### 4.5. Solution NMR spectroscopy data collection and processing

1D ^1^H solution NMR spectra were collected at 298 K for cis-aconitic acid on a 14.1 T Bruker AvanceIII and 17.4 T Bruker AvanceIII spectrometer each equipped with a TCI four-channel inverse detection H/C/N/D cryoprobe. Samples were locked to 90:10 H_2_O/D_2_O. T ^1^H 1D spectra were recorded at pH 7.5 to match protein and ligand stocks. All NMR data were processed using Bruker TopSpin and MestreMnova^33^. ^1^H chemical shifts were referenced to the water peak at 4.7 ppm. All solution spectra were analyzed using MestreMnova. ^1^H solution chemical shifts were assigned based on the published cis-aconitate and itaconate chemical shifts (Biological Magnetic Resonance Data Bank entries bmse000705 and bmse000137, respectively).

### 4.6. Enzyme Kinetic Assay

Enzyme kinetics of hACOD1 were obtained by a continuous assay observing the conversion of cis-aconitic acid to itaconic acid by ^1^H NMR. 100 mM *cis*-aconitate and citraconate stock solutions were prepared using SEC buffer. 50 nM hACOD1 samples were prepared with a final *cis*-aconitate concentration ranging from 0.25–30.0 mM to a final volume of 400 μL. ^1^H 1D spectra (32K points) of final reaction mixtures were measured at six-minute time intervals over the course of two hours. Samples prepared for citraconate inhibitory assays were measured under constant protein (50 nM) and varying substrate concentrations (20–200 μM) with citraconate concentrations ranging from 0.2 to 1.0 mM. All time points at the different conditions described were measured in triplicate.

All samples were temperature equilibrated by water-insulated heating block at 25 °C. Data was fit by Prism 10 (GraphPad) using Michaelis-Menten non-linear regression to calculate catalytic rate constant (k_cat_), maximum velocity (V_max_) and Michaelis constant (K_M_). Citraconate concentration resulting in 50% inhibition of hACOD1 activity (IC50) was determined by non-linear regression of sigmoidal dose-response. Each point represents three independent experiments error bars are +−1 SEM.

### 4.7. MD Simulations

Simulations were performed in GROMACS^34^. Each was started from apo-hACOD1 (residues 4–461) solvated in a rhombic dodecahedral water box; charges were neutralized with sodium and chloride ions and addition 150 mM NaCl was present to reproduce experimental conditions. The system was equilibrated for 1 ns in the NPT ensemble before starting production runs. Ten replicates of 200 ns with a 2 fs time step were run in parallel starting from the same equilibrated structure. Trajectories were analyzed with MDAnalysis^35^. The distance between the Cα of Tyr318 and Cα of Pro155 was measured for each protomer over every frame in the trajectories. The Cα root mean square fluctuations (RMSF) were determined by first calculating an average structure over all trajectories, aligning to this structure and calculation of the RMSF using the average structure as a reference.

## Supplementary Material

Supplement 1

## Figures and Tables

**Table 1:** Model refinement statistics.

Protein Form	Citraconate-bound form (PDB ID 9O5N)	Apo form (PDB ID9O5J)
Resolution range (Å)	34.01–1.22	33.80–1.32
No. of reflections, working set	155813	146042
No. of reflections, test set	8014	7369
Final Rcryst	15.1	15.8
Final Rfree	17.9	19.3
No. of non-H atoms	15492	15462
Protein/nucleic acid	14434	14417
Ions	2	2
Ligands	28	37
Waters	1026	1006
R.m.s. deviations from ideality		
Bonds (Å)	0.01	0.01
Angles (°)	1.896	1.823
Average B factors (Å^2^)		
Protein/nucleic acid	15.0	16.3
Ions	20.6	26.5
Ligands	19.9	46.9
Waters	28.0	29.7
Ramachandran plot (Refmac5)		
Favored regions (%)	95.31	95.53
Outliers (%)	0.22	0.22
Unmodelled/incomplete residues (%)	0	0
