# Supplementary material for: Analysis of apo and citraconate-bound hACOD1 (hIRG1) by X-ray crystallography and NMR spectroscopy: Structural insights for developing chemotherapeutic agents
Source: bioRxiv. 2025 Jun 13:2025.06.13.659517. Preprint. [Version 1] doi: 10.1101/2025.06.13.659517 (PMC12259180; doi:10.1101/2025.06.13.659517)
Supplement: Supplement 1 [file media-1.pdf]

Supplemental information

Sup. Table 1 hACOD1 Crystallization conditions

Protein form	Citraconate-bound form (9O5N)	Apo form (9O5J)
Method	Sitting drop	
Plate type	Swissci MRC3	
Temperature (°C)	20 ºC	
Protein concentration	5.1 mg/mL (15 mM citraconate)	5.9 mg/mL
Buffer composition of protein solution	10 mM HEPES pH 7.5, 150 mM NaCl, 10% glycerol, 1 mM TCEP	
Composition of reservoir solution	100 mM Tris pH 8.0, 25% PEG 4000, 200 mM CaOAc	100 mM Tris pH 8.8, 35% PEG 4000, 200 mM CaOAc
Volume and ratio of drop	200 nL, 2:1 protein:reservoir	
Volume of reservoir	40 uL	
Composition of the cryoprotectant	Mitegen LV CryoOil	
Drop setting	Formulatrix Formulator (reservoir) and NT8 (drop setting)	
Seeding	No	

Sup. Table 2 ACOD1 expression information

Source	
Source organism	Homo sapiens
DNA source	synthetic
Expression vector	pCAD29_hIRG1_4-461_pvp008[1] (addgene #124843)
Expression host	*E. coli*
Expression details	Heterologous protein expression of ACOD1 was carried out in CodonPlus (RIPL) BL21. 1 L LB cultures were inoculated to a final IPTG concentration of 500 [μM] for 18 hours at 22°C and 130 rpm agitation.
Complete amino-acid sequence of the protein produced	MASWSHPQFEKVDENLYFQ-GGGRKSITESFATAIHGLKVGHLTDRVIQ RSKRMILDTLGAGFLGTTTEVFHIASQYSKIYSSNISSTVWGQPDIRLPP TYAAFVNGVAIHSMDFDDTWHPATHPSGAVLPVLTALAEALPRSPKFSG LDLLLAFNVGIEVQGRLLHFAKEANDMPKRFHPPSVVGTLGSAAAASKF LGLSSTKCREALAIAVSHAGAPMANAATQTKPLHIGNAAKHGIEAAFLAM LGLQGNKQVLDLEAGFGAFYANYSPKVLPSIASYSWLLDQQDVAFKRF PAHLSTHWVADAAASVRKHLVAERALLPTDYIKRIVLRIPNVQYVNRPFP VSEHEARHSFQYVACAMLLDGGITVPSFHECQINRPQVRELLSKVELEY PPDNLPSFNILYCEISVTLKDGATFTDRSDTFYGHWRKPLSQEDLEEKF RANASKMLSWDTVESLIKIVKNLEDLEDCSVLTTLLKGP

Sup. Table 3 List of crystallization conditions that produced three-dimensional crystals at least 25 μm in size which lacked additives structural homologous to substrate. All conditions were screened for optimal pH, [salt], and [PEG].

**Crystallization Screen**	**Well #**	**Additive**	**Buffer**	**Precipitant**
Index	F2	TMAO [0.2M]	Tris pH 8.0 [0.1M]	PEG 2000 [20%w/v]
Index	G4	$LiSO_4$ [0.2M]	HEPES [0.1M]	PEG 3350 [25%w/v]
JCSG+	G4	TMAO [0.2M]	Tris pH 8.5 [0.1M]	PEG 2000 [20%w/v]
PACT	D7	NaCl [0.2M]	Tris pH 8.0 [0.1M]	PEG 6000 [20%w/v]
PACT	D11	$CaCl_2$ [0.2M]	TRIS pH 8.0 [0.1M]	PEG 6000 [20%w/v]
PACT	F1	NaF [0.2M]	BIS-TRIS propane pH 6.5 [0.1M]	PEG 3350 [20%w/v]
PACT	F3	NaI [0.2M]	BIS-TRIS propane pH 6.5 [0.1M]	PEG 3350 [20%w/v]
PACT	F5	$NaNO_3$ [0.2M]	BIS-TRIS propane pH 6.5 [0.1M]	PEG 3350 [20%w/v]
PACT	F6	Na formate [0.2M]	BIS-TRIS propane pH 6.5 [0.1M]	PEG 3350 [20%w/v]
Structure	C9	$MgCl_2$ [0.2M]	TRIS pH 8.5 [0.1M]	PEG 4000 [30% w/v]
Structure	D11	NaOAc [0.2M]	Tris pH 8.5 [0.1M]	PEG 4000 [30% w/v]

Sup. Table 4 X-ray data collection and reduction parameters. Values given in parenthesis are for the highest resolution shell.

Protein Form	Citraconate-bound (9O5N)	Apo (9O5J)
Diffraction source	NSLS2-17-ID-2 (FMX)	NSLS2-17-ID-1 (AMX)
Wavelength (Å)	0.979338 Å	0.920194 Å
Temperature (K)	100 K	100 K
Detector	EIGER 16M	EIGER 9M
Crystal to detector distance	158.91 mm	161.55 mm
Total rotation range	360º	360º
Rotation per image	0.2º	0.2º
Exposure time per image (s)	10 ms	10 ms
Space group	$P2_12_12$	$P2_12_12$
a, b, c (Å)	102.2, 110.4, 76.2	101.7, 110.0, 75.8
α β, γ (°)	90.0, 90.0, 90.0	90.0, 90.0, 90.0
Resolution range (Å)	33.9-1.22 (1.45-1.22) 1.32 a*, 1.44 b*, 1.506 c* Ellipsoid definition: 0.7582, 0.6970, 0.6640	33.78-1.32 (1.48-1.32) 1.39 a*, 1.43 b*, 1.51 c* Ellipsoid definition: 0.7182, 0.7010, 0.6609
Total no. of reflections	2117439 (93179)	2000190 (84956)
No. of unique reflections	155814 (7790)	146042 (7306)
Completeness (%)	Spherical: 61.4 (7.8) Ellipsoidal: 94.7 (54.9)	Spherical: 74.0 (13.1) Ellipsoidal: 95.1 (55.4)
Redundancy	13.6 (12.0)	13.7 (11.6)
$\langle I/\sigma(I) \rangle$ from merged data	10.3 (1.7)	9.8 (1.0)
CC1/2	0.66 (0.42)	1.00 (0.33)
Rmerge	0.189 (1.98)	0.204 (2.50)
Rmeas	0.202 (2.15)	0.220 (2.742)
Rpim	0.053 (0.595)	0.057 (0.789)
Overall B factor from Wilson plot (Å^2)	9.9	10.51

Sup. Fig. 1 MD trajectory analysis. A) the distance distribution between Tyr318 and Pro155, two key residues that can be used to quantify the open and closed conformational changes. The distribution is clearly bimodal, with the transition to the open form occurring at ~13.6 Å (dotted line). B) Residue fluctuations, measured as RMSF, showing the separate chains, one for each protomer in the biological dimer. In the simulations, only chain B opens. Two loops at the domain interface, A1 and A2, show increased mobility in chain B. The lid domain also moves comparatively more in chain B than in chain A. The spike at residue 298 comes from a crystal artifact where in the starting model, chain B residue 298 has a direct interaction with a crystal neighbor, influencing the initial conformation and precluding movement compared to chain A. The movement is located in a highly mobile region at the top of the lid domain, remote from the active site or domain interfaces, and is not directly correlated to hACOD1 activity.

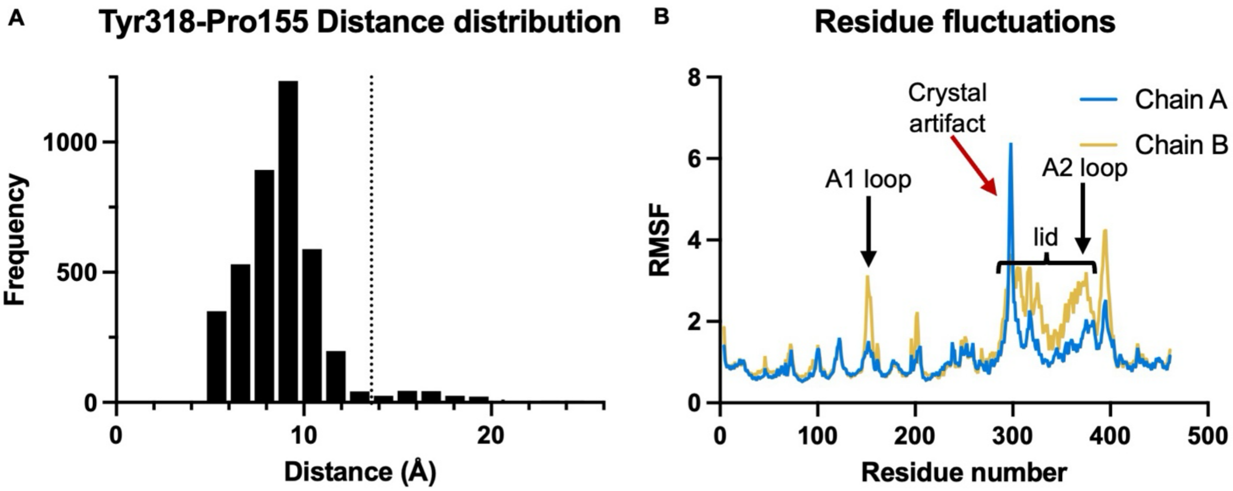

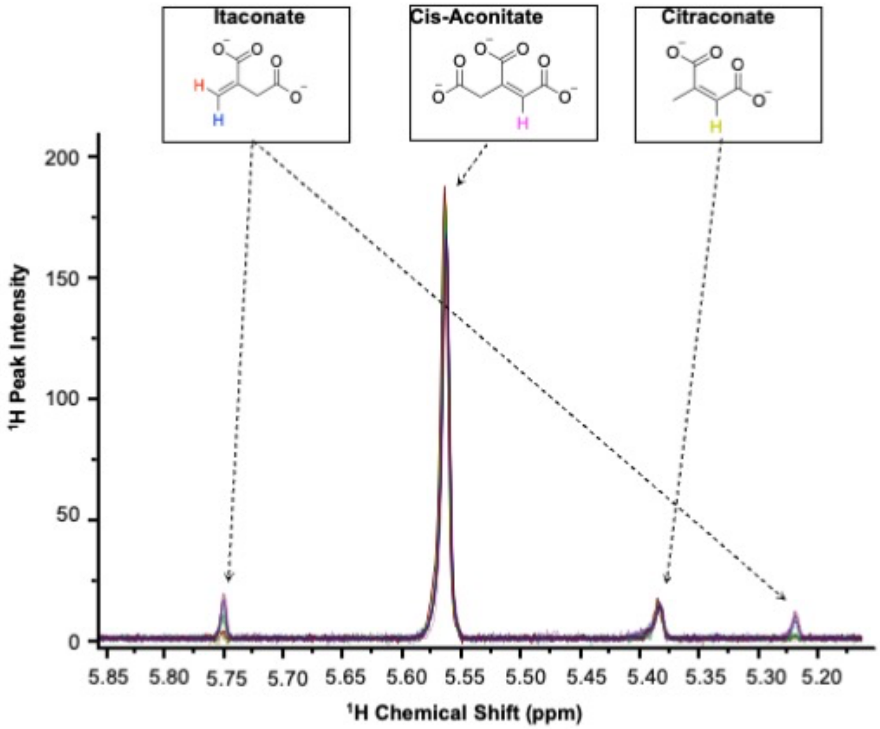

Sup. Fig. 2 ^{1}H NMR spectrum of inhibitory assay of ACOD1 by dose-dependence of citraconate.
